# Emerging Research on Gene Delivery to the Nucleus via DNA Origami

**DOI:** 10.1021/jacsau.5c00737

**Published:** 2025-08-19

**Authors:** Sierra Sterling, Yin Wei, Gaurav Arya, Carlos Castro, Yonggang Ke

**Affiliations:** † Wallace H. Coulter Department of Biomedical Engineering, Georgia Institute of Technology and Emory University, Atlanta, Georgia 30322, United States; ‡ Department of Mechanical and Aerospace Engineering, 2647 The Ohio State University, Columbus, Ohio 43210, United States; § Thomas Lord Department of Mechanical Engineering and Materials Science, Duke University, Durham, North Carolina 27708, United States

**Keywords:** DNA origami, gene delivery, rational design, targeted delivery, cellular uptake, nuclear entry

## Abstract

Structural DNA nanotechnology, a research field in which scientists use DNA as the primary material to make designer nanostructures, has experienced rapid growth in the past few decades. The continuous development of the field has produced a rich repository of impressive, complex nanostructures for applications in materials science, biological research, and therapeutics. The unprecedented programmability of DNA nanostructures, particularly DNA origami, combined with the biocompatibility and rich functionality of DNA molecules make them attractive candidates for building nanocarriers for cellular delivery. While the initial research toward this direction focused on the delivery of small molecule drugs and short nucleic acids, emerging efforts in the last two years have expanded to gene delivery by leveraging the capacity of DNA origami to fold gene sequences into compact structures amenable for cell delivery. Here, we review this exciting research direction and provide our perspective on the challenges and opportunities in this field.

## Graphical abstract

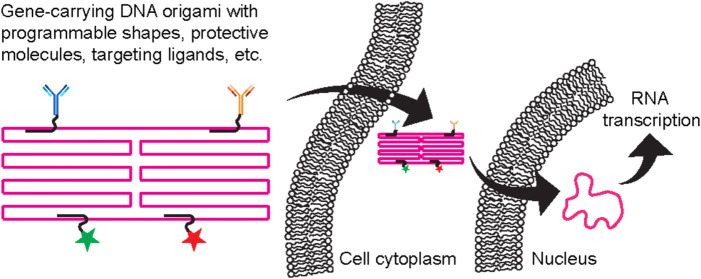

## Introduction

The advancement in genetic research has fundamentally reshaped the landscape of medicine and biotechnology, enabling unprecedented progress in understanding viral origins, treating genetic disorders, and developing targeted therapies. The ability to deliver and express custom genes in cells is essential for advancing scientific research and supports a growing array of medical and technological innovations.^1^ The delivery of cargo into the cell nucleus lies at the heart of modern genetic technology. However, efficiently targeting specific cells or tissues with genetic material remains a significant hurdle. The processes of packaging, delivering, and proper expression of nucleic acids often require tailored solutions based on specific applications. These challenges become even more complex when multiple genes must be delivered and expressed simultaneously – a capability that is increasingly becoming important for sophisticated applications such as genome or epigenome engineering, transcriptional regulation, and the design of synthetic genetic circuits.

The design of effective nanotechnology-based gene delivery methods requires careful consideration of multiple factors, including size, charge, biocompatibility, targeting specificity, and sequence. In the past few years, DNA nanotechnology has started attracting attention as gene delivery vehicles due to its unparalleled programmability in terms of physical and chemical properties and excellent biocompatibility. The field of DNA nanotechnology was invented by Seeman in the 1980s, when he proposed to use DNA as essentially a programmable biopolymer for designing nanostructures.^2^ In this unique perspective, viewing DNA simply as a polymer for making nanomaterials, the central role of DNA as the primary genetic information carrier in biological systems is often intentionally overlooked. And the focus on building nanostructures by programming interactions between DNA strands has resulted in an explosion of development of massive, sophisticated supramolecular structures.^3,4^

Since the conceptual introduction of the field, the invention of DNA origami^5^ is probably the most impactful development, both in terms of its capacity in constructing complex structures and its versatility in applications, ranging from nanofabrication, biosensing, and therapeutics.^6^ Different from many nanomaterials based on inorganic or organic molecules, DNA is an intrinsically biocompatible and biofunctional molecule, which makes it an attractive choice for diverse biological and biomedical applications. Furthermore, a typical DNA origami contains ∼ 200 staple DNA strands that can be individually modified with peptides, proteins, fluorophores, and other functional molecules for custom-designed nanostructures depending on specific uses. For example, these nanostructures can be modified with antibodies to achieve optimal targeting efficiency,^7^ or to be made more stable by coating them with other moieties, such as DNA brushes^8^ or peptoids.^9^

To date, most of the applications still use the origami design that employs a single-stranded M13 virus-derived scaffold DNA with a typical length of ∼7000–8000 nucleotides. Typically, the sequence details of DNA origami are not crucial in many applications, including biomedical applications, where the DNA origami is generally used as a carrier or pegboard with custom-designed shapes and modifications. Nonetheless, in the past few years, an exciting new research direction started to emerge – using DNA origami as a delivery vehicle for functional gene delivery.^10–14^ These advancements were enabled by previous studies that developed methods for making scaffold DNA with custom sequences. For example, Douglas and his team established a scaffold template that presented some customizability by packaging custom ssDNA sequences into phagemids for scaffold production. They were able to clone large (>3kb), insert custom sequences into phagemids and generate custom-sequenced scaffolds with lengths up to 10kb on the milligram scale.^15^ In 2019, they introduced a tool called “scaffold smith,” which produces custom scaffold sequences for DNA origami, a novel invention for the field given the numerous limitations presented by traditional ssDNA scaffolds.^16^ With this tool, the need to design 3D origami structures around generic scaffold sequences can be bypassed, and designers can design their respective structures based on sequence and not only the shape outcome. It has also opened the possibility to incorporate functional motifs directly into the scaffold and has shown how scaffold sequence can influence stability but most importantly, this approach can lead to the engineering of cross-linked, immunogenic, and modular scaffolds keen for *in vivo* applications.

While it has only recently been explored, the dual-purpose use of scaffold DNA as both the molecule for making nanostructures for delivery and the genetic information carrier is a natural advantageous feature of DNA origami. In particular, the DNA origami approach has the potential to address the delivery of large genes (i.e., ∼10 kb or more), which remains a major challenge. However, despite the promising results from a few pioneering publications so far (to be discussed in detail later), systematic and critical work remains to be carried out to understand the mechanisms of DNA-origami-based gene delivery and how design parameters influence these mechanisms to enable design optimization. This review aims to provide a brief overview on this emerging field and thus some helpful perspective to researchers interested in gene delivery to the cell nucleus via DNA origami.

Like any effective nanosystem for gene delivery, gene-carrying DNA origami needs to be able to overcome three fundamental barriers (Figure 1). First, it needs to interact with the cell membrane and get inside cells via passive or active uptake processes. Second, it must survive the cytoplasmic environment, reach the nucleus, and successfully enter it. Lastly, the gene must be converted to an active form, so it can be read and transcribed to RNA.

**Figure 1:**
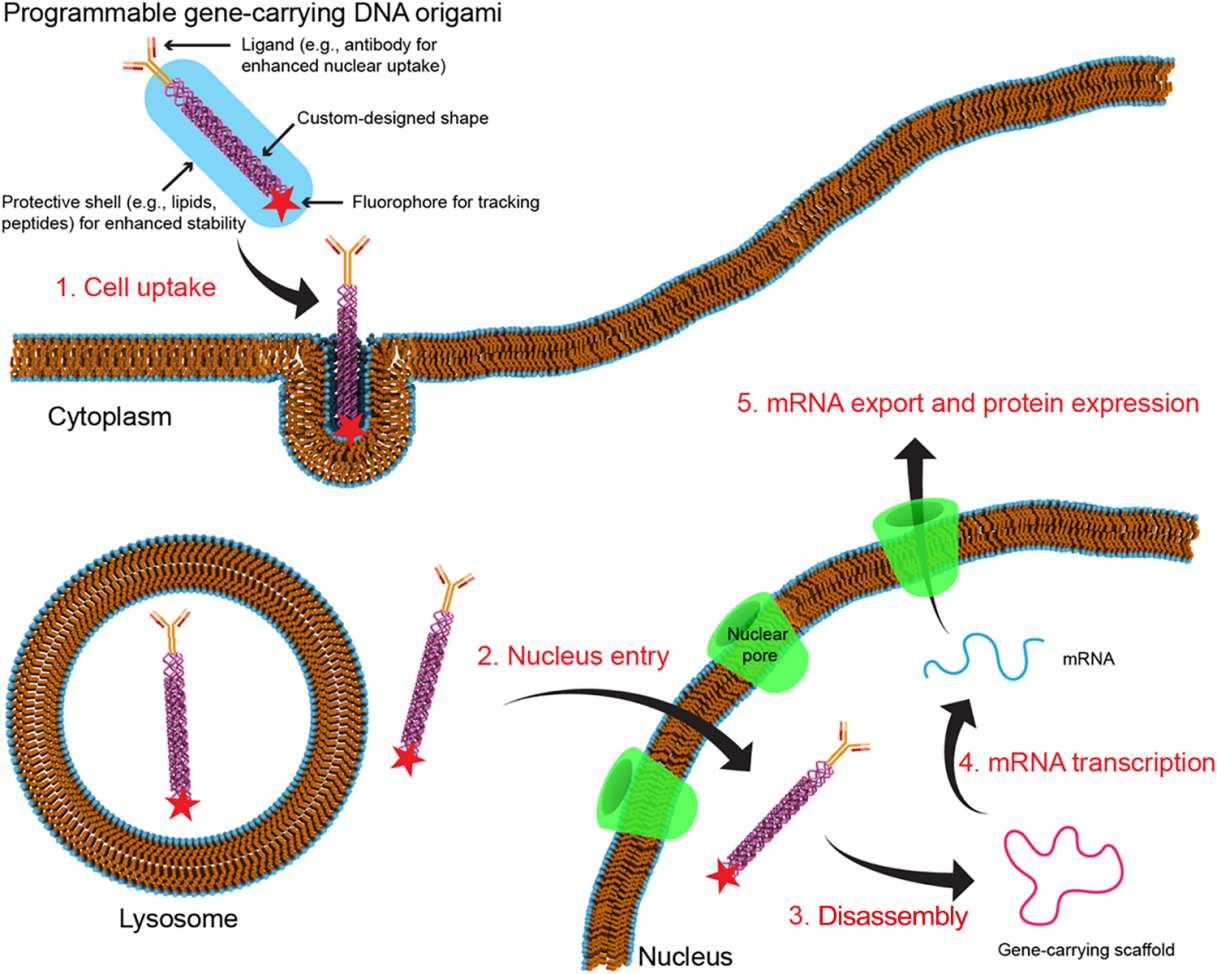
Gene delivery to the nucleus with programmable DNA origami nanostructures. The process generally involves five steps: (1) cell uptake of DNA origami that carries gene sequences; (2) entry of DNA origami into nucleus; (3) disassembly of DNA origami; (4) RNA transcription; and (5) protein expression. The scheme here is intended to illustrate the general process of how DNA origami enters the nucleus. However, the details (e.g., whether DNA origami remains intact in each step) of this process can be different, depending on DNA origami design and other factors.

## Prior Work on DNA-Origami-Based Drug Delivery into Cells

Drug delivery is one of the most promising applications of DNA origami. Thus, plenty of research has been done on investigating and improving cellular uptake, typically endocytosis, of DNA origami. These early studies provided crucial knowledge for the more recent works on DNA-origami-based gene delivery into nucleus (Figure 2). Many of these prior studies have proposed various designs and strategies for loading, delivering, and releasing molecular drugs using DNA nanostructures for cancer treatment,^17,18^ gene silencing,^19–21^ immunostimulation,^22^ and photodynamic therapy.^19,20^ These approaches typically involve functionalized DNA assemblies loaded with therapeutic agents and targeting ligands to guide them to specific sites, where the agents are then released to perform their intended functions.

**Figure 2:**
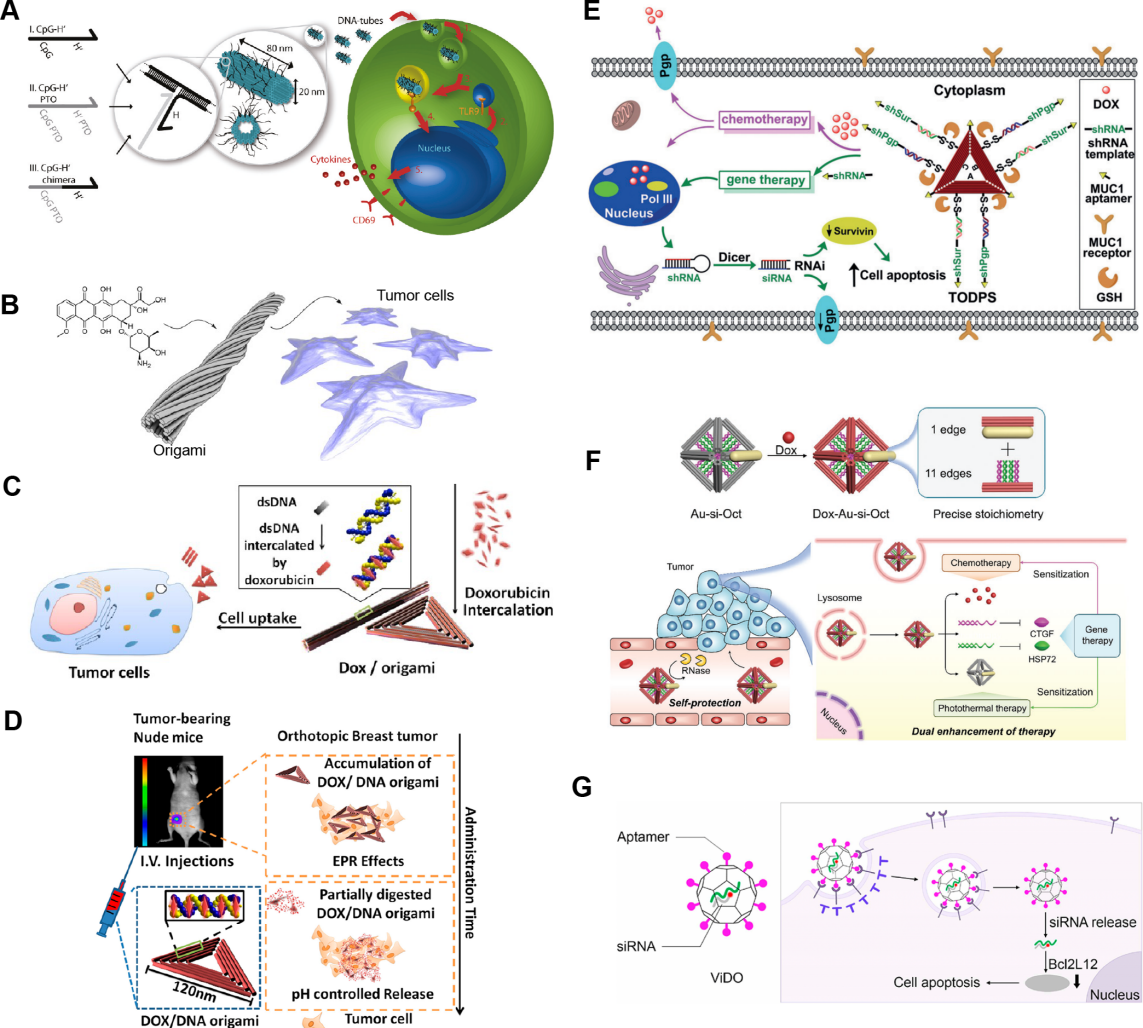
DNA origami for drug delivery into cells. (A) DNA origami nanotube for the delivery of CpG DNA cargo. Reproduced from ref 22 (copyright 2011 American Chemical Society). (B) Twisted DNA nanotube was used to load and deliver doxorubicin. Reproduced from ref 23 (copyright 2012 American Chemical Society). A triangle DNA origami was used to deliver doxorubicin to (C) tumor cells and (D) tumor-bearing mice. Reproduced from ref 17 (copyright 2012 American Chemical Society) and ref 18 (copyright 2014 American Chemical Society). (E) Targeted delivery of doxorubicin and shRNA. Reproduced with permission from ref 19 (copyright 2018 Wiley-VCH). (F) DNA origami octahedron for dual delivery of doxorubicin and self-protective siRNA. Reproduced with permission from ref 20 (copyright from 2021 Wiley-VCH). (G) Virus mimetic, truncated icosahedral DNA origami carrier for siRNA delivery. Reproduced from ref 21 (copyright 2023 American Chemical Society).

One of the earliest examples was done by the Liedl group, when they developed DNA nanotubes carrying CpG sequences to stimulate immune cells^22^ (Figure 2A). Later in 2012, two independent studies developed DNA origami structures (nanotubes and triangles) that incorporated the anticancer drug doxorubicin (DOX) via intercalation^18,23^ (Figure 2B,C). These drug-loaded constructs were successfully internalized by human breast cancer cells, leading to clear apoptotic responses. Both studies demonstrated effective drug delivery and dose-dependent therapeutic efficacy. Furthermore, in the subsequent research, Ding and colleagues investigated the in vivo performance of DNA origami drug carriers in small animals^18^ (Figure 2D) and other studies expanded to other anticancer drugs and other cancer models.^24^

Around this time, the ability to use DNA nanostructures as a drug delivery system was studied, and we learned some details about DNA nanostructure cellular uptake techniques, although much remains to be uncovered regarding structure uptake, cytoplasmic delivery, and subcellular localization and fate of the nanostructures. Zhao and colleagues successfully demonstrated tunable release properties of DNA origami delivery vehicles that undergo endocytosis, and they believed that their DNA origami slowly degraded in the endosome and that the drug is released over an extended period of time where the endosomes behave as local deposits.^23^ Several other works also pointed to origami disassembly in the endosomes. In 2011, the Liedl group demonstrated the presence of their cytosine-phosphate-guanine-containing DNA origami tubes in endosomes using a fluorescence colocalization study. While noting that more compact DNA origami structures are incorporated into the cells more easily and are more stable, their findings also strongly point to the disassembly of DNA origami tubes in the endosomes being as Toll-like receptor 9 (TLR9, which recognizes CpG oligos) are embedded into the endosomal membrane.^22^

In 2012, colocalization studies proved the localization of doxorubicin (DOX) origami structures within lysosomes following endocytosis.^17^ This was further supported by work done in 2014 by Zhang et al., who explored *in vivo* tumor uptake of DOX-occupied DNA origamis, when they noticed that drug release content increased significantly in acidic tumor regions and subcellular organelles as expected, since many anticancer drugs are weak bases, and thus pH-dependent.^18^ The slow degradation of origami structures in a low-pH environment can also potentially contribute to this process. Following these studies, work by the Castro group also demonstrated localization of DNA origami nanostructures to the lysosomes, where it is believed that drug release is initiated.^24^ Altogether, earlier DNA origami-based drug delivery methods mostly involved localization of the DNA nanostructures in the lysosome or late endosome unless organelle-specific targeting moieties were incorporated into the nanostructure design.^25^ On the other hand, DNA nanostructures have also been functionalized with pH responsive elements to enhance cytosolic drug escape^26^ and DNA origami has demonstrated enhanced stability (especially when coated with lipids, peptides, proteins, or chemical cross-linking) lasting in serum and lysates for up to 12 h.^27^

A few years later, DNA origami platforms were used for the delivery of short RNA for gene regulation. The use of nonorigami DNA nanostructures for the delivery of short interfering RNA (siRNA) was demonstrated in many earlier examples, including a DNA tetrahedron design^28^ and DNA-brick nanorods.^29^ In 2018, the triangular DNA origami, used for DOX delivery previously (Figure 2C,D), was modified for the codelivery of hairpin RNA (shRNA) and DOX^19^ (Figure 2E). The shRNAs were able to target drug resistance genes to improve the efficacy of anticancer drugs. Later, a ∼40 nm-sized DNA origami octahedron was used to deliver self-protective siRNAs, DOX, and gold nanorods.^20^ The siRNAs were loaded on the inside of the DNA origami octahedron to be protected from degradation (Figure 2F). The siRNAs downgraded targeted proteins and made the tumor cells more susceptible to chemotherapy.

Many design factors can affect the endocytosis of DNA origami and its drug delivery capability, such as structural shape, size, rigidity, stability, and surface modifications.^30^ For example, the Yang lab designed a virus-mimicking, truncated icosahedron DNA origami for improved siRNA delivery^21^ (Figure 2G). Up to 90 anchor DNA stands were placed on the inside of the DNA origami for the loading of siRNAs, and various numbers of aptamers were decorated on the outside of the structures for targeted delivery. The study showed improved uptake when the number of aptamers were increased.

## Intracellular Tracking of DNA Origami and Their Stabilization

Techniques for tracking DNA origami are critical for investigating the cellular uptake of DNA origami nanostructures and their fate inside of cells. Fluorescence imaging is the most widely used technique for tracking DNA origami. It is done by simply labeling these structures with fluorophores and monitoring their localization, distribution, and real-time dynamics within fixed or living cells. This approach provides information about how DNA origami enters cells and their general distribution inside cells, which then can be used to gain insights into the behavior of these nanostructures, such as their aggregation and interaction with subcellular organelles. However, while it is ubiquitous and useful, conventional fluorescence imaging does not provide nanoscale resolution that sometimes is necessary for understanding the stability and accurate distribution of DNA origami, which typically has a size of ∼ 100 nm. Moreover, due to background, signal overbleed, and other issues,^31^ the results of fluorescence studies need careful analysis to avoid misinterpretation. Therefore, researchers have been exploring high-resolution imaging techniques that can help us gain deeper knowledge on the process of DNA origami uptake.

High-resolution, single-molecule fluorescence imaging methods, such as single-molecule localization microscopy (SMLM), have been tested and employed for tracking DNA origami in cells. One recent example of such efforts is origamiFISH^32^ (Figure 3A). DNA origami structures were designed to carry specific probe sequences that can trigger the hybridization chain reaction (HCR), a commonly used signal amplification technique, to achieve single-molecule resolution with high sensitivity. Besides fluorescence imaging, other imaging methods, such as transmission electron microscopy (TEM), have also been used for tracking DNA origami. TEM provides exceptionally high resolution, compared to fluorescence imaging, but is generally not considered suitable for tracking DNA origami in cells due to low contrast between DNA origami and cellular molecules. To solve this, gold nanoparticles are often used to label and track DNA origami nanostructures. For example, multiple 10 nm gold nanoparticles were placed on a DNA origami nanorod to generate a signature barcode arrangement for tracking DNA origami cellular uptake (with high-resolution details such as positions and orientations of DNA origami), intracellular lifetime, and endosome escape^33^ (Figure 3B).

**Figure 3:**
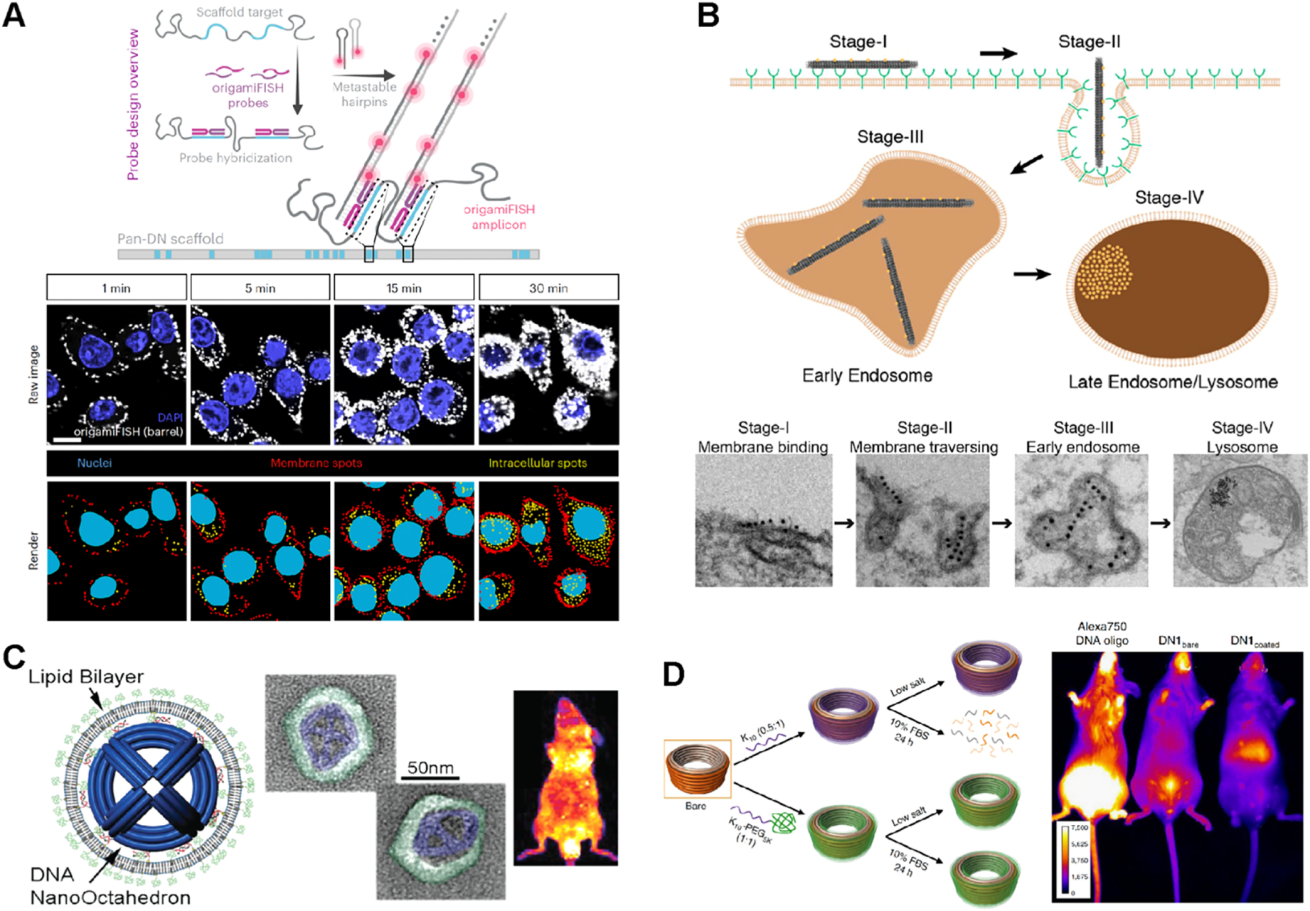
Methods for high-resolution, intracellular tracking and stabilization. (A) OrigamiFISH method allows high-resolution, single-molecule imaging of DNA nanostructures in fixed cells. Reproduced with permission from ref 32 (copyright 2024 Springer Nature). (B) 10 nm gold nanoparticles were attached to a DNA nanorod for imaging and tracking DNA origami during cell uptake. Reproduced from ref 33 (copyright 2018 American Chemical Society). (C) PEG-modified lipid bilayer-coated DNA origami octahedron improves the structural stability for in vivo delivery and imaging. Reproduced from ref 34 (copyright 2014 American Chemical Society). (D) Coating DNA origami ring with polylysine-PEG stabilizes the structures under low salt condition and improve stability in vivo. Reproduced with permission from ref 35 (copyright 2017 Springer Nature).

Such high-resolution imaging techniques are highly valuable for studying DNA origami cellular delivery in detail. Nonetheless, it is worth noting that high-resolution methods generally are slow or cannot be used in live cell imaging. Therefore, they are often not suitable to studying dynamics of DNA origami in living cells; a synergetic use of high-resolution imaging methods, regular fluorescence imaging, and other methods is necessary for gaining a more comprehensive understanding of cellular uptake and intracellular behaviors of DNA origami.

Imaging techniques for tracking DNA origami will undoubtedly be useful for research on DNA-origami-based gene delivery. To reduce unwanted toxicity and to increase the lifetime of DNA origami for drug delivery, previous research has also developed many methods for stabilization of DNA origami nanostructures. Some of these methods included cross-linking DNA strands or used modified DNA,^30,36^ which can render the gene sequences unreadable by RNA polymerase and thus may not be used in DNA-origami-based gene delivery. However, other methods, especially those involving the coating of DNA origami with protecting molecules, could be used to improve the structural stability of DNA origami without compromising the activity of gene sequences. Several groups have found that the cell internalization efficiency and biostability of DNA origami can be enhanced via their encapsulation with proteins, lipids, and polymers. For example, the Kostaininen group coated a rectangular DNA origami with cowpea chlorotic mottle virus capsid proteins and showed that the encapsulated nanostructures exhibited more than 10-fold internalization efficiency to HEK293 cells.^37^ The Shih lab coated a DNA origami octahedron with poly­(ethylene glycol) (PEG) modified lipid bilayer and demonstrated increased in vivo stability in mice^34^ (Figure 3C). Later, they developed another PEG-coating method by using positive-charged polylysine peptide modified with PEG polymer to cover many DNA origami nanostructures, including a DNA origami ring, which showed superior stability in destabilizing conditions and strong bioavailability in mice^35^ (Figure 3D).

## DNA-Origami-Based Gene Delivery

In principle, DNA origami is considered an attractive system for gene delivery to nuclei because it can fold long DNA strands into small, compact, nuclease-resistant nanostructures, which can potentially improve delivery efficiency in both cell uptake and nucleus entry. Furthermore, DNA origami may help address challenges of immunogenicity associated with viral delivery systems.^38^ Off-targeting effects can also be addressed by decorating origami with ligands to target specific types. However, it is not until recent years when we started to see a few studies that showed promising results of gene delivery to cells (Figure 4). There are probably two main reasons for this seemingly delayed research development. The first is the practical difficulty of making custom-designed scaffold DNA strands that carry the intended genes and other necessary segments for RNA transcription. The second is that the research on improving DNA origami stability and endocytosis efficiency, which was discussed in the previous sections, had finally reached a more mature stage, where the scientists had sufficient knowledge and tools to tackle this more challenging research direction. Table 1 summarizes recent studies on DNA-origami-based gene delivery.

**Figure 4:**
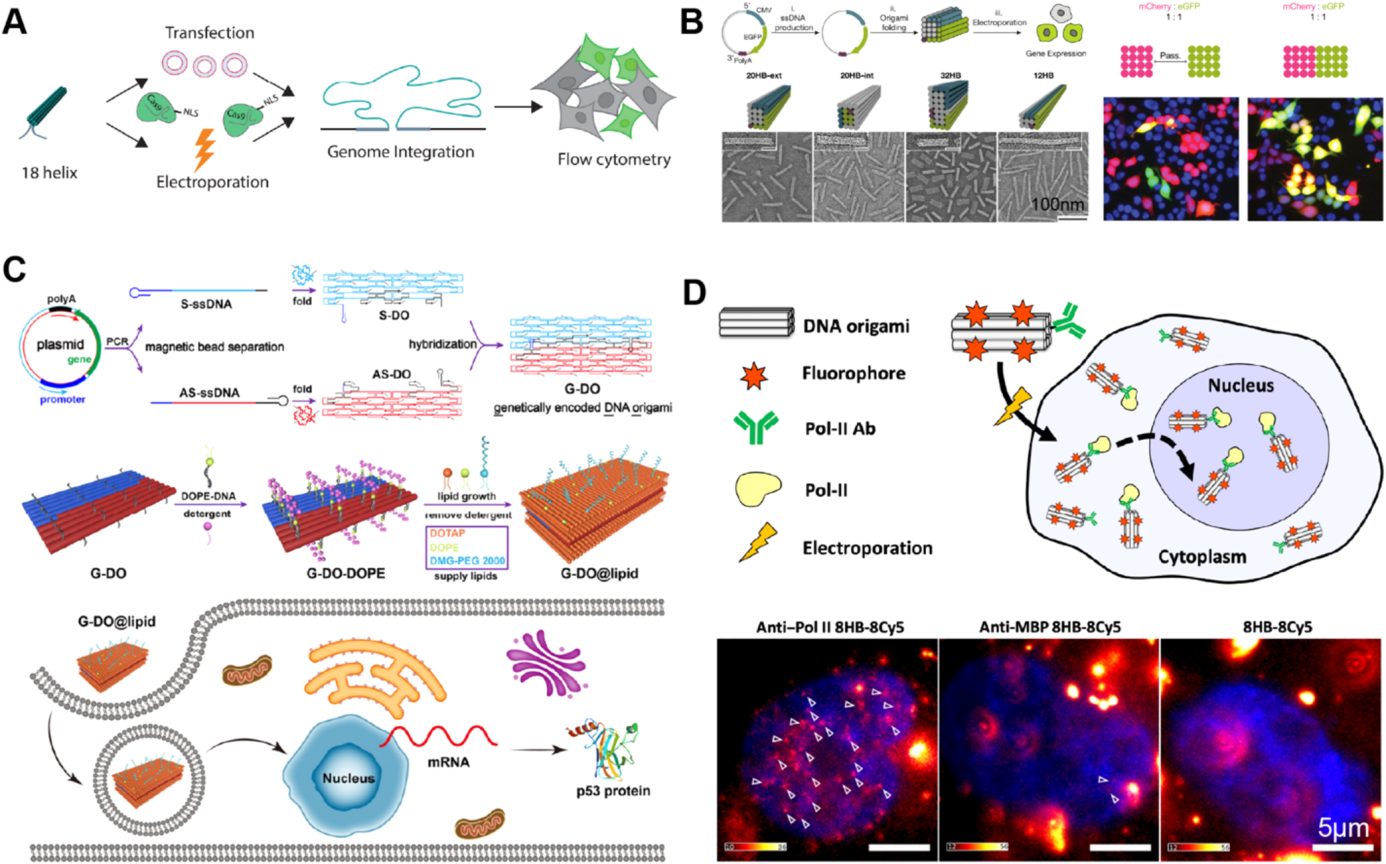
DNA origami for delivery to nucleus and gene expression. (A) Gene incorporated into the scaffold strand of DNA origami was integrated into the genome by using CRISPR-Cas9. Reproduced with permission from ref 10 (copyright 2022 Oxford University Press). (B) Single-stranded DNA scaffolds containing eGFP/mCherry genes were folded into nanorod-shaped DNA origami for delivery to the nucleus and gene expression. Reproduced with permission from ref 11 (copyright 2023 Springer Nature). (C) Sense strand and antisense strand of the p53 protein gene were incorporated into two single-stranded scaffolds, which were then folded into a DNA origami that contains both sense strand and antisense strand for nucleus delivery and protein expression. Reproduced from ref 12 (copyright 2023 American Chemical Society). (D) A piggybacking strategy, where DNA origami was decorated with Pol II targeting antibody, showed significant improvement of nucleus entry of DNA origami (white arrows indicate the fluorescence signal on DNA origami inside the nucleus). Reproduced with permission from ref 39 (copyright 2024 The American Association for the Advancement of Science).

**Table 1:** Summary of Recent Investigations on DNA-Origami-Based Gene Delivery

publication	origami design	gene delivered	nucleus targeting	cell type
ref [Bibr ref10], 2022	18-helix nanotube	GFP, mCherry	Cas9 target sequence	HEK293T, K562
ref [Bibr ref11], 2023	20-, 32-, 12-helix nanorods	eGFP, mCherry		HEK293T, K562
ref [Bibr ref12], 2023	rectangle	eGFP, p53		HEK293T, HeLa
ref [Bibr ref14], 2023	20-helix nanorod	mCherry	SV40 DTS sequence	HEK293T

Pioneering work in 2022, done in collaboration between the Doudna lab, Castro lab, and others, first demonstrated the ability to fold and deliver gene sequences in gene encoding DNA origami structures that could enter cell nuclei and ultimately be expressed by cells into fluorescent proteins^10^ (Figure 4A). This study focused on using gene encoding DNA origami as a template for homology-directed repair, leveraging CRISPR-Cas9 to target specific homology sites. The DNA origami was designed as a tube with homology sequences extending from one end of the tube in close proximity. Results showed that eGFP could be expressed in model cell lines and primary human T cells. The work further demonstrated integration with CRISPR-Cas9 with homology domains on DNA origami enabled integration of genes at specific genome sites and that multiple genes, in this case a multigene cassette encoding two fluorescent proteins, could be delivered and expressed. Finally, while most of the work was carried out by delivering nanostructures via electroporation, experiments were also performed incorporating gene encoding DNA origami with virus-like particles (VLPs), where genes folded into DNA origami were expressed with higher efficiency than the single-stranded template, providing an interesting path for further exploration.

In 2023, work by the Dietz group adopted a different strategy. Using a single-stranded DNA contains the eGFP gene and a CMV promoter as the scaffold, they constructed a group of nanorod-shaped DNA origami, which was delivered to mammalian cells via electroporation^11^ (Figure 4B). Their study showed that these DNA origami could enter cell nuclei and express eGFP, presumably suggesting the DNA origami can disassemble, release the scaffold, and initiate subsequent RNA transcription. They also showed multiple genes (e.g., eGFP and mCherry) could be incorporated together in DNA origami scaffold for successful gene delivery to nuclei. Their results challenge previous findings that single-stranded DNA plasmids have much lower activity in RNA transcription than their double-stranded counterparts.^40,41^ In the same year, another study by the Song lab investigated the gene expression efficacy of single-stranded DNA plasmids and showed that they generally possess reasonably good activities in most of the cell lines that were tested.^13^

In another research lab, Ding and his colleagues tried to address the concern of low gene expression efficiency of single-stranded DNA plasmid with a different approach – codelivery of both the sense and antisense DNA strands^12^ (Figure 4C). Also in 2023, they used a clear design strategy that used two single-stranded DNA scaffolds, one containing the sense gene sequence and the other containing antisense gene sequences, to fold single DNA origami as the delivery system. The origami was also coated with PEG to improve its biostability. Their promising results showed successful expression of the p53 protein. However, the study does not provide enough information to provide a clear answer to the question about what really happened to the sense DNA scaffold and antisense scaffold. Do they effectively engage in the RNA transcription process independently or do they work synergistically (e.g., forming double-stranded plasmid)?

To move this research forward, more studies are needed to answer important questions regarding mechanisms related to gene delivery and expression via DNA origami. Based on common knowledge and available experimental data, several studies on DNA origami-based gene delivery all hypothesized similar pathways for mRNA transcription, after gene-carrying DNA origami enters nucleus.^10–14^ It is suggested that the DNA origami first needs to disassemble into single-stranded form, which will be converted to double-stranded DNA, primarily by cellular mechanisms for DNA repair. The double-stranded DNA template was then used for mRNA transcription and subsequent protein expression.

A recent study focused on the specific delivery of DNA origami nanostructures into live cell nuclei^39^ (Figure 4D). Inspired by prior work to target antibodies to the nucleus,^42^ this work leverages existing nuclear import traffic mechanisms to deliver DNA origami nanostructures into live cell nuclei after electroporation. Instead of adding a nuclear targeting signal directly to the DNA origami, the DNA origami was designed to bind to a cytoplasmic target, which is then naturally trafficked to the nucleus. To this end, DNA origami was conjugated with an anti-Pol-II antibody. After electroporation, the antibody attached to an RNA Polymerase II (Pol II) subunit in the cytoplasm; the antibody-conjugated DNA origami could then piggyback with the Pol II complex to get into the cell nucleus. Live cell imaging showed successful nuclear localization of Cy5-labeled DNA origami when it is conjugated with anti-Pol-II antibody. The work demonstrated an approach to achieve efficient nucleus targeting, offering a promising pathway to use DNA origami to deliver genes into cell nuclei and more broadly to leverage intracellular transport mechanisms to traffic DNA origami to specific cellular compartments. This work also revealed that large DNA origami nanostructures suffered aggregation in the cytoplasm and had difficulty entering cell nuclei, highlighting the importance of careful design of the shape and surface modification of DNA origami for effective intracellular delivery.

In more recent studies, the use of DNA nuclear targeting sequences (DTS) as well as nuclear localization signal (NLS) peptides has also been exploited for the delivery of DNA origami into the nucleus. Liedl and colleagues designed DNA origami constructs using a custom scaffold embedded with multiple DTS sequences; this enabled the nuclear delivery of DNA nanostructures via active import through importin-mediated pathways.^14^ In another study, researchers intercalated NLS peptides into DNA origami structures and observed not only a marked enhancement in nuclear import, but also successful gene expression in human cells.^43^ Both studies represent significant advancements, and ongoing efforts are continuing to explore DNA origami-based delivery systems through the investigation of mammalian cellular transport. Future studies should consider evaluating this technique in large animal models, including nonhuman primates, to support its development for nuclear gene delivery applications.

## Challenges and Outlook

This review highlights the evolving potential of DNA nanotechnology as a highly programmable and biocompatible platform for gene therapy. Key breakthroughs in cellular and nuclear delivery demonstrate how structural features, functionalization, and design innovations enhance stability, uptake, and therapeutic efficacy. it is important to note that some limitations of DNA nanostructure-based deliveries remain elusive and will soon be addressed. The challenges associated with achieving efficient and targeted delivery of DNA nanostructures to the nucleus include endosomal entrapment and insufficient endosomal release, elicitation of an immune recognition response, structural complexity of hosting targeting moieties, and restriction to diffusion across nuclear membranes.

DNA nanotechnology offers several advantages over viral and synthetic cellular delivery systems. One of its primary strengths lies in the precise spatial organization it enables: drugs, siRNA, proteins, aptamers, and targeting moieties can be positioned at defined sites on the nanostructure, enhancing both drug accumulation and targeting specificity. Because DNA nanostructures are composed entirely of synthetic DNA, they can be chemically modified to minimize immune activation, rendering them highly biocompatible. This contrasts with viral and nonviral delivery systems, which vary significantly in immunogenicity. To avoid triggering innate immune responses – such as activation of cGAS, STING, IFI16, and other DNA sensors, researchers have modified DNA scaffolds chemically and minimized or eliminated CpG motifs from their designs. Encapsulation of nanostructures in biocompatible carriers, promoting endosomal enclosure, and designing compact origami with minimal exposed DNA are all strategies used to reduce immune activation.^22^

Various DNA origami designs have demonstrated higher cellular uptake efficiencies. Additionally, ligands and targeting moieties have been shown to facilitate endosomal escape and subcellular localization to the nucleus, lysosomes, mitochondria, and specific cell types – further emphasizing the programmability of these systems. It is also worth highlighting the implementation of logic gate actuation within cells by using DNA nanostructures. Multiple researchers have explored this concept, leveraging DNA logic gates as a platform for parallel, highly controlled therapeutic delivery responsive to intracellular triggers. A system with both high targeting specificity and logic-based responsiveness opens the door to constructing synthetic cellular machinery.

Breakthroughs in DNA-origami-based gene delivery will also benefit from in-depth mechanistic investigation, which would likely require detailed colocalization studies. While fluorescence imaging remains the most widely used method for live-cell tracking of DNA origami, advanced imaging techniques, such as gold nanoparticle barcoding, transmission electron microscopy (TEM), and super-resolution methods, have also been used to track membrane binding, endosomal entry, conformational changes, and trafficking of DNA nanostructures.^33,44^ However, currently there is no technique that can probe conformational changes in DNA nanostructures in real time inside living cells. Achieving this would require super-resolution imaging capable of visualizing site-specific mechanical reporters within the nanostructure. To accomplish this, endosomal escape is necessary to allow unfolding of the structure within the cytoplasm. Future innovations may combine molecular tagging and advanced imaging to enable real-time tracking of the behavior of DNA nanostructures, especially their interactions with transcriptional machinery. Once optimized, such a technique could transform DNA nanotechnology and expand its therapeutic applications dramatically.

Besides experimental investigation, computational modeling can play a vital role in the design and delivery of DNA origami nanostructures into cells and ultimately into the nucleus. An obvious application is in the design phase – designing the origami of shapes and sizes that ensure efficient transport across the cell membrane and through the nuclear pore complex. Previous studies have shown that certain origami geometries are preferentially taken up by cells and must stay within specific size ranges to achieve effective delivery.^45^ Molecular dynamics (MD) simulations, especially using efficient coarse-grained models like oxDNA,^46^ have proven to be invaluable for testing the stability of different DNA nanostructure designs^39^ or for predicting their conformational dynamics in the case of reconfigurable DNA devices.^47,48^ Coarse-grained MD simulations of DNA origami that also integrate coarse-grained models of cell membranes such as the Martini force field^49^ could further provide insights into how the shape and size of DNA origami affects their uptake, how membrane bending or receptor binding might occur, and how different designs could improve endocytosis or avoid endosomal trapping.^50^ Increasingly, AI and machine learning tools are starting to be applied toward predicting the shape of DNA origami structures,^51^ assessing their structural stability under physiological conditions,^52^ and automated high-throughput characterization of DNA origami structures and their yield based on microscopy images.^53,54^ This is a rapidly evolving area with many exciting developments on the horizon.

Despite experimental progress, there is a limited understanding of how DNA nanostructures are transported within the cytoplasm and into the nuclei. Intermolecular interactions with intracellular proteins, RNA, and other DNA molecules can potentially disrupt the conformation of DNA origami inside cells. All-atom MD simulations could be a useful tool here for predicting the effects of such interactions, though very little work has been done along this front due to the complexity of the cytoplasm environment. Moreover, all-atom models would struggle to capture the dynamics of transport processes that involve large length and time scales. What is needed are coarse-grained models, but currently no such model exists that can simultaneously and accurately model DNA and proteins, though recent modeling efforts are beginning to address this issue.^55,56^ AI/machine learning methods could again be very useful here, as evidenced by their recent success in identification of protein and DNA characteristics that promote protein corona formation on DNA nanostructures in vivo.^57^

Effectively translating DNA nanotechnology from the laboratory to clinical applications remains a fundamental challenge in its own right. Translating DNA nanotechnology from the lab to clinical applications involves overcoming not only technical hurdles but also regulatory challenges that are often complex, multitiered, and evolving. These challenges affect all stages of product development, from design to clinical trials and market approval. First, it is difficult to determine the regulatory classification of DNA as there can be some uncertainty in whether it should fall under the regulations for the drug, biologic, or device category. This is only a peek into the requirements yet to be uncovered in clinical applications to successfully introduce DNA nanostructures in clinical settings. Being that DNA nanotechnology remains uncommon in clinical applications, lack of various requirements, such as standardized manufacturing and quality control protocols, good manufacturing standards, quantitative validation of nuclear entry, and targeting efficiency, can all complicate clinical designs and effectively hinder the transition of DNA nanotechnology delivery systems into clinical applications. In addition to these considerations, the yield of DNA origami currently achievable is adequate only for laboratory-scale applications and not for mass manufacturing. Manual purification methods, such as gel electrophoresis, are also commonly used but are unsuitable for large-scale clinical production and must be reevaluated. Similarly, conventional quality control techniques, including TEM and AFM, are not easily scalable. When all of these factors are taken into account, the costs associated with this technique remain relatively high, highlighting the need for more cost-effective approaches to improve its feasibility.

We believe future advancements in DNA nanotechnology are poised to revolutionize nuclear delivery systems by enabling greater precision, programmability, and responsiveness to cellular cues. Innovations such as mechanoactivated devices, multifunctional nanostructures, and strategically designed constructs may significantly improve targeting efficiency and nuclear import. Achieving these breakthroughs will require close collaboration among nanotechnologists, molecular biologists, and clinicians to ensure that laboratory innovations are effectively translated into viable tools.

## References

[1] Boti M. A., Athanasopoulou K., Adamopoulos P. G., Sideris D. C., Scorilas A. (2023). Recent Advances
in Genome-Engineering Strategies. Genes.

[2] Seeman N. C. (1982). Nucleic
acid junctions and lattices. J. Theor. Biol..

[3] He Y., Ye T., Su M., Zhang C., Ribbe A. E., Jiang W., Mao C. (2008). Hierarchical
self-assembly of DNA into symmetric supramolecular polyhedra. Nature.

[4] Ke Y., Ong L. L., Shih W. M., Yin P. (2012). Three-dimensional structures
self-assembled from DNA bricks. Science.

[5] Rothemund P. W. K. (2006). Folding
DNA to create nanoscale shapes and patterns. Nature.

[6] Wang P. F., Meyer T. A., Pan V., Dutta P. K., Ke Y. G. (2017). The Beauty
and Utility of DNA Origami. Chem.

[7] Shaw A., Hoffecker I. T., Smyrlaki I., Rosa J., Grevys A., Bratlie D., Sandlie I., Michaelsen T. E., Andersen J. T., Hogberg B. (2019). Binding to
nanopatterned antigens
is dominated by the spatial tolerance of antibodies. Nat. Nanotechnol..

[8] Yang Y., Lu Q., Huang C. M., Qian H., Zhang Y., Deshpande S., Arya G., Ke Y., Zauscher S. (2021). Programmable Site-Specific
Functionalization of DNA Origami with Polynucleotide Brushes. Angew. Chem., Int. Ed..

[9] Wang S. T., Gray M. A., Xuan S., Lin Y., Byrnes J., Nguyen A. I., Todorova N., Stevens M. M., Bertozzi C. R., Zuckermann R. N., Gang O. (2020). DNA origami protection
and molecular
interfacing through engineered sequence-defined peptoids. Proc. Natl. Acad. Sci. U.S.A..

[10] Lin-Shiao E., Pfeifer W. G., Shy B. R., Saffari
Doost M., Chen E., Vykunta V. S., Hamilton J. R., Stahl E. C., Lopez D. M., Sandoval Espinoza C. R. (2022). CRISPR-Cas9-mediated nuclear transport and genomic integration of
nanostructured genes in human primary cells. Nucleic Acids Res..

[11] Kretzmann J. A., Liedl A., Monferrer A., Mykhailiuk V., Beerkens S., Dietz H. (2023). Gene-encoding DNA origami
for mammalian cell expression. Nat. Commun..

[12] Wu X., Yang C., Wang H., Lu X., Shang Y., Liu Q., Fan J., Liu J., Ding B. (2023). Genetically Encoded
DNA Origami for Gene Therapy In Vivo. J. Am.
Chem. Soc..

[13] Tang L., Tian Z., Cheng J., Zhang Y., Song Y., Liu Y., Wang J., Zhang P., Ke Y., Simmel F. C., Song J. (2023). Circular single-stranded DNA as switchable
vector for gene expression in mammalian cells. Nat. Commun..

[14] Liedl A., Griessing J., Kretzmann J. A., Dietz H. (2023). Active Nuclear Import of Mammalian Cell-Expressible DNA Origami. J. Am. Chem. Soc..

[15] Nafisi P. M., Aksel T., Douglas S. M. (2018). Construction
of a novel phagemid
to produce custom DNA origami scaffolds. Synth.
Biol..

[16] Shen K., Flood J. J., Zhang Z., Ha A., Shy B. R., Dueber J. E., Douglas S. M. (2024). Engineering an *Escherichia
coli* strain for production of long single-stranded
DNA. Nucleic Acids Res..

[17] Jiang Q., Song C., Nangreave J., Liu X., Lin L., Qiu D., Wang Z. G., Zou G., Liang X., Yan H., Ding B. (2012). DNA origami as a carrier
for circumvention of drug resistance. J. Am.
Chem. Soc..

[18] Zhang Q., Jiang Q., Li N., Dai L., Liu Q., Song L., Wang J., Li Y., Tian J., Ding B., Du Y. (2014). DNA origami as an in vivo drug delivery
vehicle for cancer therapy. ACS Nano.

[19] Liu J., Song L., Liu S., Zhao S., Jiang Q., Ding B. (2018). A Tailored DNA Nanoplatform
for Synergistic RNAi-/Chemotherapy of
Multidrug-Resistant Tumors. Angew. Chem., Int.
Ed..

[20] Xu T., Yu S., Sun Y., Wu S., Gao D., Wang M., Wang Z., Tian Y., Min Q., Zhu J. J. (2021). DNA Origami Frameworks Enabled Self-Protective siRNA
Delivery for Dual Enhancement of Chemo-Photothermal Combination Therapy. Small.

[21] Shi Q., Wu Y., Xu Y., Bao M., Chen X., Huang K., Yang Q., Yang Y. (2023). Virus Mimetic Framework
DNA as a Non-LNP Gene Carrier for Modulated Cell Endocytosis and Apoptosis. ACS Nano.

[22] Schüller V. J., Heidegger S., Sandholzer N., Nickels P. C., Suhartha N. A., Endres S., Bourquin C., Liedl T. (2011). Cellular immunostimulation by CpG-sequence-coated
DNA origami structures. ACS Nano.

[23] Zhao Y. X., Shaw A., Zeng X., Benson E., Nystrom A. M., Hogberg B. (2012). DNA origami delivery
system for cancer
therapy with tunable release properties. ACS
Nano.

[24] Halley P. D., Lucas C. R., McWilliams E. M., Webber M. J., Patton R. A., Kural C., Lucas D. M., Byrd J. C., Castro C. E. (2016). Daunorubicin-Loaded
DNA Origami Nanostructures
Circumvent Drug-Resistance Mechanisms in a Leukemia Model. Small.

[25] Shishparenok A. N., Furman V. V., Zhdanov D. D. (2023). DNA-Based Nanomaterials as Drug Delivery
Platforms for Increasing the Effect of Drugs in Tumors. Cancers.

[26] Song L., Ho V. H. B., Chen C., Yang Z., Liu D., Chen R., Zhou D. (2013). Drug Delivery: Efficient, pH-Triggered
Drug Delivery Using a pH-Responsive DNA-Conjugated Gold Nanoparticle
(Adv. Healthcare Mater. 2/2013). Adv. Healthcare
Mater..

[27] Kim J., Narayana A., Patel S., Sahay G. (2019). Advances in intracellular
delivery through supramolecular self-assembly of oligonucleotides
and peptides. Theranostics.

[28] Lee H., Lytton-Jean A. K., Chen Y., Love K. T., Park A. I., Karagiannis E. D., Sehgal A., Querbes W., Zurenko C. S., Jayaraman M. (2012). Molecularly self-assembled
nucleic acid nanoparticles for targeted in vivo siRNA delivery. Nat. Nanotechnol..

[29] Rahman M. A., Wang P., Zhao Z., Wang D., Nannapaneni S., Zhang C., Chen Z., Griffith C. C., Hurwitz S. J., Chen Z. G. (2017). Systemic
Delivery of
Bc12-Targeting siRNA by DNA Nanoparticles Suppresses Cancer Cell Growth. Angew. Chem., Int. Ed..

[30] Wang Y., Lu X., Wu X., Li Y., Tang W., Yang C., Liu J., Ding B. (2022). Chemically
modified DNA nanostructures for drug delivery. Innovation.

[31] Lacroix A., Vengut-Climent E., de Rochambeau D., Sleiman H. F. (2019). Uptake and Fate of Fluorescently Labeled DNA Nanostructures
in Cellular Environments: A Cautionary Tale. ACS Cent. Sci..

[32] Wang W. X., Douglas T. R., Zhang H., Bhattacharya A., Rothenbroker M., Tang W., Sun Y., Jia Z., Muffat J., Li Y., Chou L. Y. T. (2024). Universal, label-free,
single-molecule visualization of DNA origami nanodevices across biological
samples using origamiFISH. Nat. Nanotechnol..

[33] Wang P., Rahman M. A., Zhao Z., Weiss K., Zhang C., Chen Z., Hurwitz S. J., Chen Z. G., Shin D. M., Ke Y. (2018). Visualization of the Cellular Uptake
and Trafficking of DNA Origami Nanostructures in Cancer Cells. J. Am. Chem. Soc..

[34] Perrault S. D., Shih W. M. (2014). Virus-inspired membrane
encapsulation
of DNA nanostructures to achieve in vivo stability. ACS Nano.

[35] Ponnuswamy N., Bastings M. M. C., Nathwani B., Ryu J. H., Chou L. Y. T., Vinther M., Li W. A., Anastassacos F. M., Mooney D. J., Shih W. M. (2017). Oligolysine-based
coating protects
DNA nanostructures from low-salt denaturation and nuclease degradation. Nat. Commun..

[36] Gerling T., Kube M., Kick B., Dietz H. (2018). Sequence-programmable
covalent bonding of designed DNA assemblies. Sci. Adv..

[37] Mikkilä J., Eskelinen A. P., Niemela E. H., Linko V., Frilander M. J., Torma P., Kostiainen M. A. (2014). Virus-encapsulated
DNA origami nanostructures for cellular delivery. Nano Lett..

[38] Lucas C. R., Halley P. D., Chowdury A. A., Harrington B. K., Beaver L., Lapalombella R., Johnson A. J., Hertlein E. K., Phelps M. A., Byrd J. C., Castro C. E. (2022). DNA Origami Nanostructures
Elicit Dose-Dependent Immunogenicity and Are Nontoxic up to High Doses
In Vivo. Small.

[39] Roozbahani G. M., Colosi P. L., Oravecz A., Sorokina E. M., Pfeifer W., Shokri S., Wei Y., Didier P., DeLuca M., Arya G. (2024). Piggybacking
functionalized
DNA nanostructures into live-cell nuclei. Sci.
Adv..

[40] Wang J., Xie J., Lu H., Chen L., Hauck B., Samulski R. J., Xiao W. (2007). Existence of transient functional double-stranded DNA intermediates
during recombinant AAV transduction. Proc. Natl.
Acad. Sci. U.S.A..

[41] Ping H., Liu X., Zhu D., Li T., Zhang C. (2015). Construction and Gene
Expression Analysis of a Single-Stranded DNA Minivector Based on an
Inverted Terminal Repeat of Adeno-Associated Virus. Mol. Biotechnol..

[42] Conic S., Desplancq D., Ferrand A., Fischer V., Heyer V., Reina San
Martin B., Pontabry J., Oulad-Abdelghani M., Babu N. K., Wright G. D. (2018). Imaging of native transcription
factors and histone phosphorylation at high resolution in live cells. J. Cell Biol..

[43] Oh C. Y., Kaur H., Tuteja G., Henderson E. R. (2024). DNA origami
drives gene expression in a human cell culture system. Sci. Rep..

[44] Eilers Y., Ta H., Gwosch K. C., Balzarotti F., Hell S. W. (2018). MINFLUX monitors
rapid molecular jumps with superior spatiotemporal resolution. Proc. Natl. Acad. Sci. U.S.A..

[45] Rajwar A., Shetty S. R., Vaswani P., Morya V., Barai A., Sen S., Sonawane M., Bhatia D. (2022). Geometry of
a DNA Nanostructure Influences Its Endocytosis: Cellular Study on
2D, 3D, and in Vivo Systems. ACS Nano.

[46] Snodin B. E. K., Randisi F., Mosayebi M., Sulc P., Schreck J. S., Romano F., Ouldridge T. E., Tsukanov R., Nir E., Louis A. A., Doye J. P. K. (2015). Introducing
improved structural properties and salt dependence into a coarse-grained
model of DNA. J. Chem. Phys..

[47] Shi Z., Castro C. E., Arya G. (2017). Conformational
Dynamics of Mechanically
Compliant DNA Nanostructures from Coarse-Grained Molecular Dynamics
Simulations. ACS Nano.

[48] Shi Z., Arya G. (2020). Free energy landscape
of salt-actuated reconfigurable
DNA nanodevices. Nucleic Acids Res..

[49] Marrink S. J., Risselada H. J., Yefimov S., Tieleman D. P., de Vries A. H. (2007). The MARTINI force
field: coarse grained model for biomolecular
simulations. J. Phys. Chem. B.

[50] Sousa de Almeida M., Susnik E., Drasler B., Taladriz-Blanco P., Petri-Fink A., Rothen-Rutishauser B. (2021). Understanding nanoparticle endocytosis
to improve targeting strategies in nanomedicine. Chem. Soc. Rev..

[51] Truong-Quoc C., Lee J. Y., Kim K. S., Kim D. N. (2024). Prediction
of DNA origami shape using graph neural network. Nat. Mater..

[52] Zubia-Aranburu, J. ; Gardin, A. ; Paffen, L. ; Tollemeto, M. ; Alberdi, A. ; Termenon, M. ; Grisoni, F. ; Patino Padial, T. Predicting DNA origami stability in physiological media by machine learning, 2025.

[53] Wang Y., Jin X., Castro C. (2023). Accelerating
the characterization
of dynamic DNA origami devices with deep neural networks. Sci. Rep.

[54] Chen C., Nie J., Ma M., Shi X. (2023). DNA Origami
Nanostructure Detection and Yield Estimation Using Deep Learning. ACS Synth. Biol..

[55] DeLuca M., Sensale S., Lin P. A., Arya G. (2024). Prediction
and Control in DNA Nanotechnology. ACS Appl.
Bio Mater..

[56] Procyk J., Poppleton E., Sulc P. (2021). Coarse-grained nucleic
acid-protein model for hybrid nanotechnology. Soft Matter.

[57] Huzar J., Coreas R., Landry M. P., Tikhomirov G. (2025). AI-Based Prediction
of Protein Corona Composition on DNA Nanostructures. ACS Nano.

